# HLA DR Genome Editing with TALENs in Human iPSCs Produced Immune-Tolerant Dendritic Cells

**DOI:** 10.1155/2021/8873383

**Published:** 2021-05-20

**Authors:** Yoo-Wook Kwon, Hyo-Suk Ahn, Jin-Woo Lee, Han-Mo Yang, Hyun-Jai Cho, Seok Joong Kim, Shin-Hyae Lee, Heung-Mo Yang, Hyun-Duk Jang, Sung Joo Kim, Hyo-Soo Kim

**Affiliations:** ^1^Strategic Center of Cell and Bio Therapy for Heart, Diabetes & Cancer, Biomedical Research Institute, Seoul National University Hospital, Seoul 03080, Republic of Korea; ^2^Stem Cell Convergence Research Center, Korea Research Institute of Bioscience and Biotechnology (KRIBB), 125 Gwahak-ro, Yuseong-gu, Daejeon 34141, Republic of Korea; ^3^Cardiovascular Center & Department of Internal Medicine, Seoul National University Hospital, Seoul 03080, Republic of Korea; ^4^ToolGen, Inc., #1204, Byucksan Digital Valley 6-cha, 219 Gasan Digital 1-ro, Geumcheon-gu, Seoul 08501, Republic of Korea; ^5^SK Chemical, Life Science Business, Clinical Research Team, Gyeonggi-do, 13494, Republic of Korea; ^6^GenNBio, Inc., 422, Teheran-ro, Gangnam-gu, Seoul 06193, Republic of Korea; ^7^Molecular Medicine and Biopharmaceutical Sciences, Graduate School of Convergence Science and Technology, Seoul National University, Seoul 03080, Republic of Korea

## Abstract

Although human induced pluripotent stem cells (iPSCs) can serve as a universal cell source for regenerative medicine, the use of iPSCs in clinical applications is limited by prohibitive costs and prolonged generation time. Moreover, allogeneic iPSC transplantation requires preclusion of mismatches between the donor and recipient human leukocyte antigen (HLA). We, therefore, generated universally compatible immune nonresponsive human iPSCs by gene editing. Transcription activator-like effector nucleases (TALENs) were designed for selective elimination of HLA DR expression. The engineered nucleases completely disrupted the expression of HLA DR on human dermal fibroblast cells (HDF) that did not express HLA DR even after stimulation with IFN-*γ*. Teratomas formed by HLA DR knockout iPSCs did not express HLA DR, and dendritic cells differentiated from HLA DR knockout iPSCs reduced CD4^+^ T cell activation. These engineered iPSCs might provide a novel translational approach to treat multiple recipients from a limited number of cell donors.

## 1. Introduction

Since iPSCs are autologous or customizable pluripotent stem cells derived from somatic cells, they overcame two major hurdles of ES cells, namely, ethical controversies and immune rejection following transplantation in patients. However, this approach has several limitations with respect to genetic mutations in patients in addition to the expense of treatment in addition to prolonged durations required for the generation and manipulation of autologous iPSCs. The timely availability of the desired cells, when required by multiple patients, is often not feasible. In 2008, a national project was conceived in Japan to resolve this problem. It was estimated that up to 30 HLA homozygous iPS cell lines, which show a three-locus (HLA-A, -B, and -DR) match in 82.2% of the Japanese population, would be available. Increasing the number HLA homozygous iPS cell lines to 50 could potentially enable 90.7% of the Japanese population to be covered by this cell bank [1]. However, this system is not adequate to meet global needs. Thus, an alternative for the reduction or prevention of immunological rejection is required. To address this issue, we focused on generating immune tolerant human iPSCs that would obtain immune nonresponsiveness through gene editing. Several reports have demonstrated that mismatches at the HLA DR locus reveal the most significant impact on the development of an alloimmune response against transplanted organs such as the heart, kidney, and lung [2–7]. In this study, we designed transcription activator-like effector nucleases (TALENs) for selective elimination of HLA DR expression, and we used surrogate reporters to enrich cells containing nuclease-induced mutations via MACS. After TALEN-encoding plasmid transfection, four independent single cell-derived HLA DR knockout clones are obtained. All clones were HLA DR negative and did not respond to the stimulation of IFN-*γ*. The generation of human iPSCs was achieved by the retroviral vector-mediated transduction of four specific transcription factors: Oct4, Klf4, Sox2, and c-Myc (OKSM), into HLA DR knockout HDFs.

The dendritic cells (DCs) are of pivotal importance in both innate and adaptive immunities. DCs integrate danger signals and downmodulatory cues to direct an adaptive immune response [8] This study demonstrated that HLA DR knockout iPSCs could differentiate to DCs with efficiency comparable to that of wild-type iPSCs. DCs from HLA DR knockout iPSCs did not activate allogenic T cells whereas DCs from WT-iPSCs did. These findings indicated that the derivatives of HLA DR knockout iPSCs could reduce CD4^+^ T cell activation and therefore provide a new option to overcome immune rejection in allogenic stem cell therapy.

## 2. Materials and Methods

### 2.1. Cell Culture, Nuclear Transfection, and Magnetic Sorting

Human dermal fibroblasts (HDFs) were grown in Dulbecco's modified Eagle's medium high glucose containing 10% fetal bovine serum, penicillin, and streptomycin. Plasmid DNAs containing the left and right TALEN-targeting HLA DR and reporter plasmids used in this study were purchased from ToolGen, Inc. To introduce indels at the target site, 1 × 10^6^ HDF cells were transfected with a total 9 *μ*g of TALEN plasmids and reporters (4 *μ*g plasmid encoding left TALEN, 4 *μ*g plasmid encoding right TALEN, and 1 *μ*g of reporter plasmid) using an AMAXA Human Dermal Fibroblast Nucleofector kit (Amaxa Biosystems, Lonza, Switzerland) according to the manufacturer's protocol. Two days after the DNA delivery, the cells were isolated with a magnetic bead-conjugated H-2K^k^ antibody (MACSelect K^k^ microbeads; Miltenyi Biotec, Germany) for 20 min at 4°C. Labeled cells were separated on a column (MACS LS column; Miltenyi Biotec, Germany) according to the manufacturer's instructions and seeded as individual cells into a 96-well plate. After 2 weeks, each clone was transferred into 6-well plates for further expansion (Supplementary Figure S1A).

### 2.2. T7E1 Assay, Fluorescence PCR, and Sequencing Analysis

Genomic DNA from individual KO candidate clones was isolated using a NucleoSpin Tissue Kit (Macherey-Nagel, Germany) according to the manufacturer's instructions. T7E1 assay was performed as previously described [9]. Briefly, we performed PCR to amplify the region containing the target site using the primer 5′-CCTGGGTTTGCAGAGAGCAGAC-3′ and 5′-CCTACACTTCTCCTCTTCC CAGA-3′. The PCR products including the TALEN target site were denatured by heating and annealed to form heteroduplex DNA, which was treated with 5 units of T7 endonuclease 1. Fluorescence PCR analysis was performed using 5′ FAM-labeled primer. The PCR amplicons were analyzed at Macrogen, Inc. For sequencing analysis, PCR amplicons containing TALEN-induced small indel mutations were purified using the Gel extraction kit (Zymo Research, USA) and cloned into the vector using a cloning kit (Elpis Bio, Korea). Several colonies were picked and sequenced by Sanger sequencing (Cosmogentech, Inc., Korea).

### 2.3. Generating HLA DR KO-iPS Cells

Moloney-based retroviral vectors (pMXs) containing complementary human DNAs of Oct4, Sox2, Klf4, and c-Myc were obtained from Addgene. Human dermal fibroblasts (HDF) were seeded at 5 × 10^4^ cells per 6-well plates, one day before transduction with 8 *μ*g/ml Polybrene (Sigma-Aldrich, USA). And the cells were transduced with a concentrated OSKM retrovirus. Next day, the virus-containing medium was replaced with a HDF medium containing 50 *μ*g/ml vitamin C. 6 days after transduction, fibroblasts were harvested by trypsinization and replated at 8 × 10^4^ cells per 6-well plates on a mitomycin C- (Sigma-Aldrich, USA) treated STO feeder layer. On the following day, the medium was replaced with DMEM/F12 (Invitrogen, USA) supplemented with 20% knockout serum replacement (Invitrogen, USA), 2 mM L-glutamine (Invitrogen, USA), 1% nonessential amino acids (Invitrogen, USA), 0.1 mM *β*-mercaptoethanol (Invitrogen, USA), 1% penicillin/streptomycin, and 10 ng/ml bFGF (R&D). 50 *μ*g/ml of vitamin C (Sigma-Aldrich, USA) and 0.5 mM valproic acid (VPA, Sigma-Aldrich, USA) were added till the appearance of iPSC-like cells [10]. The medium was changed every day. Colonies were picked up mechanically and transferred into mitomycin C-treated STO feeder layers.

### 2.4. Alkaline Phosphatase and Immunochemistry

Alkaline phosphatase (AP) activity was determined using an alkaline phosphatase detection kit (BCIP/NBT, Promega, USA). For immunocytochemical staining, iPSCs were fixed with 4% paraformaldehyde for 15 min at room temperature. And the cells were permeabilized with 0.1% Triton X-100 and then blocked with 1% bovine serum albumin (Amresco, Inc., USA). Staining was carried out using primary anti-Oct4 (1 : 100, Santa Cruz, USA), anti-Nanog (1 : 100, Santa Cruz, USA), anti-SSEA4 (1 : 100, Santa Cruz, USA), antibiotinylated CD45 (1 : 40; R&D Systems, USA), and anti-HLA DR (1 : 100; Abcam, UK), and samples were incubated overnight at 4°C. Appropriate Alexa Fluor® dye-conjugated secondary antibodies were as follows: donkey anti-mouse Alexa 555 (1 : 200; Invitrogen, USA), donkey anti-mouse Alexa 488 (1 : 200; Invitrogen, USA), and DAPI (1 : 5000, Invitrogen, USA) were used for nuclear counterstaining. Images were obtained using a confocal microscope (LSM 510 Meta; Carl Zeiss, Germany).

### 2.5. Teratoma Formation and Immunohistochemistry

A total of 3 × 10^6^ cells of iPSCs were injected subcutaneously in the testis of NSG mice (NOD scid IL-2 receptor gamma chain knockout). 12 weeks later, the tumors were excised and fixed in 4% paraformaldehyde overnight. Each tissue was embedded in paraffin, and tissue sections were stained with hematoxylin-eosin.

### 2.6. *In Vitro* Differentiation of HLA DR Wild-Type and Knockout iPSCs into Dendritic Cell

The protocol for induction of differentiation of iPSCs into the dendritic cell (DC) was modified from a previously published paper [11]. iPSC colonies were transferred onto a 6-well plate coated with growth factor-reduced Matrigel (BD Bioscience, USA) in mTeSR1 medium (Stemcell Technologies, Canada). The cells were differentiated with medium supplemented with BMP4 (80 ng/ml, R&D Systems, USA) for 4 days. The mTeSR1 medium (Stemcell Technologies, Canada) was replaced by a StemPro-34 serum-free medium (Thermo Fisher Scientific, USA) containing 2 mM glutamax (Invitrogen, USA), hVEGF (80 ng/ml, PeproTech, USA), hSCF (100 ng/ml, PeproTech, USA), and basic FGF (25 ng/ml, R&D Systems, USA). The cytokines in the StemPro-34 medium (Thermo Fisher Scientific, USA) were replaced by another cytokine cocktail composed of hSCF (50 ng/ml, PeproTech, USA), IL-3 (50 ng/ml, PeproTech, USA), hFlt-3 ligand (50 ng/ml, PeproTech, USA), M-CSF (50 ng/ml, PeproTech, USA), and thrombopoietin (5 ng/ml, PeproTech, USA) on day 6. Thereafter, the medium was changed on day 10. The cytokines in StemPro-34 medium were switched to a monocytic lineage differentiation cytokine cocktail supplemented with a hFlt-3 ligand (50 ng/ml, PeproTech, USA), M-CSF (50 ng/ml, PeproTech, USA), and GM-CSF (25 ng/ml, PeproTech, USA) on day 15. The medium was changed every 3 days. The CD14^+^ cells were positively sorted by an autoMACS Pro (Miltenyi Biotec, USA) with CD14-conjugated beads (MicroBeads) on days 25−28. For differentiation into matured DCs, CD14^+^ cells were seeded in a 6-well plate with Ultra-Low Attachment Surface (Corning, USA) and cultured in RPMI medium (Invitrogen, USA) supplemented with 10% FBS, GM-CSF (50 ng/ml, PeproTech, USA), and IL-4 (50 ng/ml, PeproTech, USA) for 5 days. And the maturation of iPSC-DCs was induced by adding lipopolysaccharides (100 mg/ml, Sigma-Aldrich, USA) and TNF-*α* (20 ng/ml, PeproTech, USA) for the last 2 of the 7 days.

### 2.7. Mixed Leukocyte Reactions

PBMCs (peripheral blood mononuclear cells) were prepared from a healthy donor (contributed by Kwon YW) and purchased from ATCC, USA, after Ficoll (Ficoll-Paque Plus, GE Healthcare Life Sciences, USA) gradient centrifugation. For CD4^+^ T cell isolation from PBMC, CD4^+^ T cells were purified with the CD4^+^ T cell isolation kit (Miltenyi Biotec, USA) as described in the manufacturer's protocol. Briefly, non-CD4^+^ T cells were magnetically labeled by using a cocktail of biotin-conjugated antibodies and anti-biotin microbeads. Labeled cells were removed by using an LS MACS column (Miltenyi Biotec, USA). A total of 1 × 10^4^ cells of stimulator cells (WT-iPSC-derived DC, KO-iPSC-derived DC, and PBMC-derived DC) were treated with MMC and were incubated with the responder PBMCs or CD4^+^ T cells at a ratio of 1 : 10 in a 96-well plate in RPMI-1640 (Invitrogen, USA) supplemented with 10% FBS. After 5 days of coculture, BrdU incorporation was determined according to the manufacturer's instructions (Roche, Switzerland).

### 2.8. May-Grunwald-Giemsa Staining

WT- and KO-DC were seeded onto glass slides by Cytospin 4 (Thermo Scientific, USA) and stained with Giemsa stain and modified solution (Sigma-Aldrich, USA) and analyzed by light microscopy.

### 2.9. Statistical Analysis

The results are expressed as means ± standard deviations (SD). The differences between the groups were compared by the unpaired *t*-test. *P* values ≤ 0.05 were considered statistically significant. All statistical analyses were performed using GraphPad Prism 5 software (GraphPad Software).

## 3. Results

### 3.1. Design and Validation of TALEN for Knocking Out HLA DR in Human Dermal Fibroblasts

**Figure 1:**
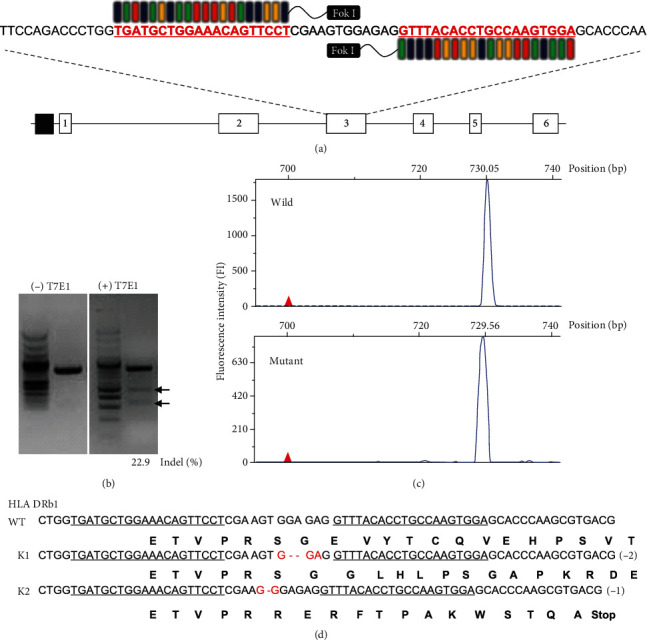
Design of the TALEN for disrupting HLA DR expression on HDF and characterization of HLA DR knockout cells. (a) Schematic of TALEN binding sites within the HLA DR region. The red and underlined nucleotides highlight the anticipated binding sites for the left and right arms of TALEN. (b) Levels of HLA DR genetic disruption determined by the T7E1 assay. The lower bands (arrows) mark the digestion products, indicating TALEN-mediated gene modification. (c) Fluorescent intensity of wild-type and mutant fibroblast cells are different after transfection of the TALEN-encoding plasmids. (d) Alignment of the genomic sequences of wild type and mutant clone at the TALEN recognition site.

To generate universally compatible and immune nonresponsive human iPSCs, we knocked out HLA DR in human fibroblasts using TALENs, which have fewer off-target events than Cas9 and are more maneuverable than ZFNs [12]. With the complexity of HLA family genes with similar sequences, TALENs that recognize more extended target sequences (36~40 bp) have a significant advantage over the widely used CRISPR/Cas9 system, which recognizes much shorter sequences (22 bp) [13]. We aligned the sequences of DRB1 subtypes most commonly observed within the Korean population [14] and screened for a target site, which can be cleaved with the same pair of TALENs. We found a potential TALEN target site in exon 3 with a sequence variation only within a spacer region among the aligned alleles (Figure 1(a)). We designed 3 different pairs (L1, L2, L3 and R1, R2, R3) of TALEN, and the most efficient TALEN pairs were selected from them. We tested all 3 pairs of TALEN with T7E1 assay and found L3 and R3 to be most efficient (Supplementary Figure S1A, B). Next, we confirmed the efficiency of this pair of TALENs with a surrogate reporter system in HEK293 cells [9]. The reporter plasmid that encodes a monomeric RFP- (mRFP-) enhanced GFP (eGFP) fusion protein, and the programmable HLA DR target site was inserted between the DNA sequences encoding mRFP and eGFP (Supplementary Figure S2). Because the eGFP sequence is fused to the mRFP sequence out of frame, this reporter plasmid expresses mRFP but not eGFP without cotransfection with TALEN. Both reporter plasmid and the nuclease are transfected in HEK293 cells, and the nuclease cleaves DNA at the target site in the reporter to generate a double-strand break resulting in error-prone nonhomologous end-joining indel mutations that give rise to frameshift mutations and result in the expression of both mRFP and eGFP. We observed only the L1 and R1 pairs of TALEN-transfected cells to be positive for both RFP and GFP fluorescence in HEK293 cells (Supplementary Figure S3). From these results, we concluded that the L1 and R1 TALEN pairs were able to target the right position and generate a double-strand break in HEK293 cells. However, the TALENs may not be adequately active in target cells that adversely affect the generation of cells containing TALEN-induced mutations. To enrich cells in which the endogenous target sequence is modified by the TALEN, we used a magnetic reporter, in which the H-2K^k^ surface marker is expressed by frame-shifting indel formation in the reporter target sequence [15]. Three days after the cotransfection of the reporter plasmid and a plasmid encoding HLA DR targeting TALEN into HDF cells, we isolated H-2K^k^ expressing cells by magnetic separation using a magnetic bead-conjugated anti-H-2K^k^ antibody. To obtain single-cell clones from the TALEN-modified HDF pool to assess the effect of knocking out the HLA DR expression, we performed a single cell culture after magnetic sorting-based separation (Supplementary Figure S1B). We tested these clones by T7E1 and fluorescence PCR. Those clones that were suggested to contain mutations by the above tests were further sequenced to confirm indels. We obtained a total of 4 clones that were further sequenced and confirmed to have deletions within the expected TALEN-targeting sites in HLA-DR, leading to frameshift leading and premature termination of translation (Figures 1(b)–1(d) and Supplementary Figure 4-6).

### 3.2. Loss of HLA DR Protein Expression on HLA DR KO Fibroblast after Genetic Editing with TALENs

**Figure 2:**
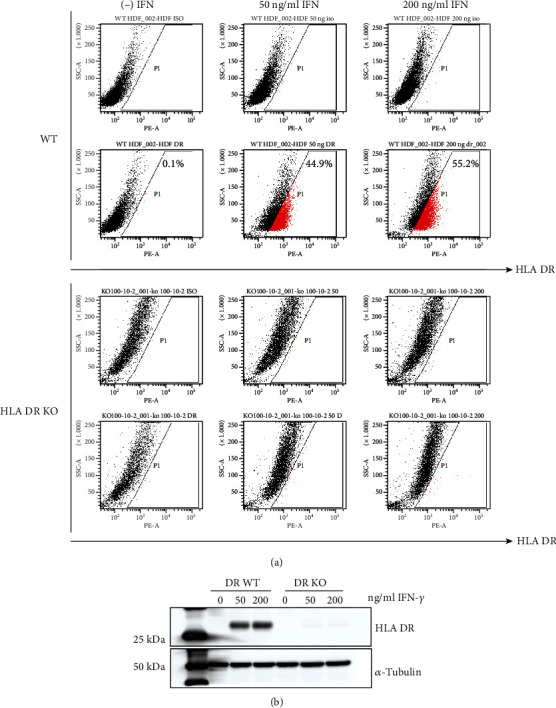
Loss of HLA DR expression on HLA DR knockout fibroblast after genome editing with TALENs. Loss of HLA DR protein expression in HLA DR knockout cells. HLA DR protein expression in wild-type and HLA DR knockout fibroblasts was analyzed by (a) FACS analysis and (b) western blotting. IFN-*γ* (50 ng/ml or 200 ng/ml) was treated for 5 days on both cell types.

Because the normal level of the HLA DR expression in HDFs is low compared to that in hematopoietic cells, we exposed the HDFs to proinflammatory cytokines known to augment HLA DR levels. The addition of IFN-*γ* increased the expression of HLA DR in parental HDFs (Figure 2(a), top). In contrast, HDFs with HLA DR knocked out by TALEN did not express HLA DR proteins even when incubated with high concentrations of IFN-*γ* (200 ng/ml) (Figure 2(a) bottom). Functional elimination of HLA DR was further confirmed by probing HDFs with HLA DR knocked out using the HLA DR antibody. In western blotting, HLA DR protein expression after induction by IFN-*γ* was not detectable in HDFs with HLA DR knocked out (Figure 2(b)). We tested the other three unique HLA DR knockout clones (150-5-1, 100-15-4, and 100-10-2), and they did not express HLA DR even after IFN-*γ* stimulation (Supplementary Figure 4-6). Together, these data suggested that genome editing using TALEN could efficiently generate HLA DR knockout fibroblasts.

### 3.3. Characterization of iPS Cells Derived from WT and HLA DR KO Fibroblast Cells

**Figure 3:**
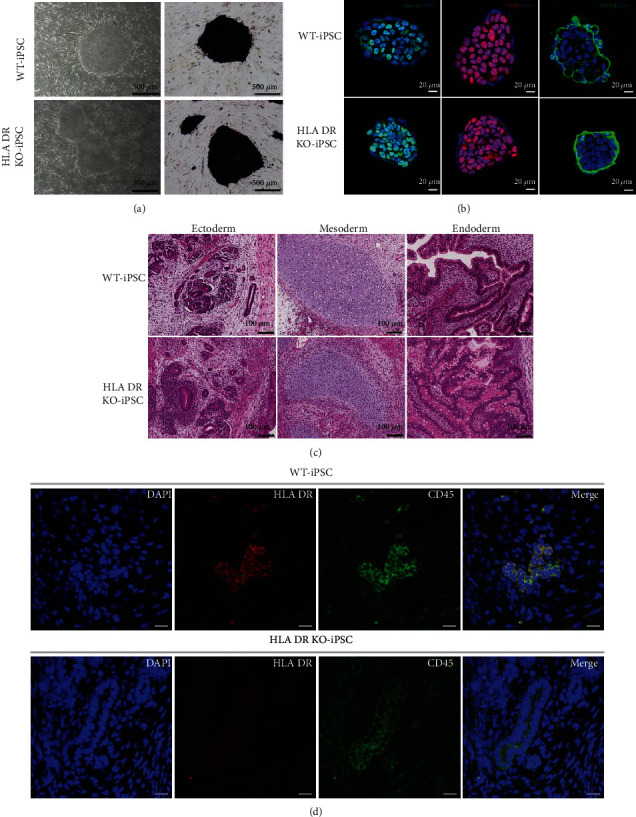
Characterization of wild-type and HLA DR knockout iPSCs. Both wild-type and HLA DR knockout iPSCs show similar stemness potential. (a) Alkaline phosphatase staining of WT and HLA DR knockout iPS colonies. (b) Immunostaining of iPSCs for pluripotency-related genes Nanog, Oct4, and SSEA4. (c) Teratoma formation showed that wild-type and HLA DR knockout iPSCs differentiated into three germ layers of tissues in vivo (scale bars: 200 *μ*m) and (d) compared the expression of HLA DR expression in CD45-positive tissues (scale bars: 200 *μ*m). Only wild type teratomas showed HLA DR expression in CD45-positive tissues.

It was more challenging to generate HLA DR knockout iPSCs than wild-type iPSCs. We made several attempts under various conditions to optimize iPSC generation including incubation with vitamin C and valproic acid (VPA), p53 knockdown, and optimization of the ES culture media before we succeeded in generating a single colony of HLA DR knockout iPSCs (Supplementary Table S1). To stabilize the iPSC colonies, we passaged iPSCs 7 times (55 days after transduction of 4 factors) and carried out several assays to characterize them. Both wild-type and mutant iPSCs exhibited comparably strong ALP activity (Figure 3(a)). Next, to verify the pluripotency of HLA DR knockout iPSCs, we checked the expression level of pluripotency-related proteins in the established HLA DR-targeted iPSCs. Immunocytochemistry revealed that both the wild-type and HLA DR knockout iPSCs were positive for pluripotency markers such as Oct4, Nanog, and SSEA4 (Figure 3(b)). When wild-type and HLA DR knockout iPSCs were injected into nonobese diabetic/severe combined immune-deficient (NOD/SCID) mice, the well-defined teratomas were observed after 2 months. Tissues derived from both wild-type and HLA DR knockout teratomas contained the various derivatives of the three germ layers, indicating the development of well-differentiated teratomas (Figure 3(c)). There were no differences in hematoxylin-eosin (HE) staining in either section. These results suggested that HLA DR knockout did not affect iPSC pluripotency.

To investigate the expression of HLA DR in tissues differentiated from human iPSCs, we performed immunocytochemistry for CD45, a common leukocyte marker. We performed double staining with CD45 and HLA DR antibodies based on the principle that normal CD45-positive tissue should express HLA DR. Interestingly, tissues of teratomas from wild-type iPSC revealed CD45 and HLA DR double-positivity. However, teratomas derived from HLA DR knockout iPSCs showed positive for CD 45 while negative for HLA DR (Figure 3(d)). This indicated that the tissue which generated from HLA DR knockout iPSCs might gain immune tolerance.

### 3.4. Generation of Dendritic Cells with Immune Tolerance

Previous reports have pointed out that hESCs and iPSCs do not express HLA class II, even when forming EBs or under IFN-*γ* induction [16–18] To investigate whether HLA DR knockout iPSCs express HLA class I, we treated both wild-type and knockout iPSCs with IFN-*γ*. As shown in Supplementary Figure 7, HLA class I was expressed, irrespective of IFN-*γ* treatment, in both wild-type and knockout iPSCs. However, for cell replacement therapy, differentiated rather than pluripotent stem cells are transplanted into the lesion. HLA class II molecules including HLA DR are expressed constitutively in antigen-presenting cells, thymic epithelial cells, and endothelial cells that affect transplantation efficiency. To ensure the functional disruption of HLA DR, we differentiated wild-type and knockout iPSCs into dendritic cells which have antigen-presenting and T cell stimulation functions.

**Figure 4:**
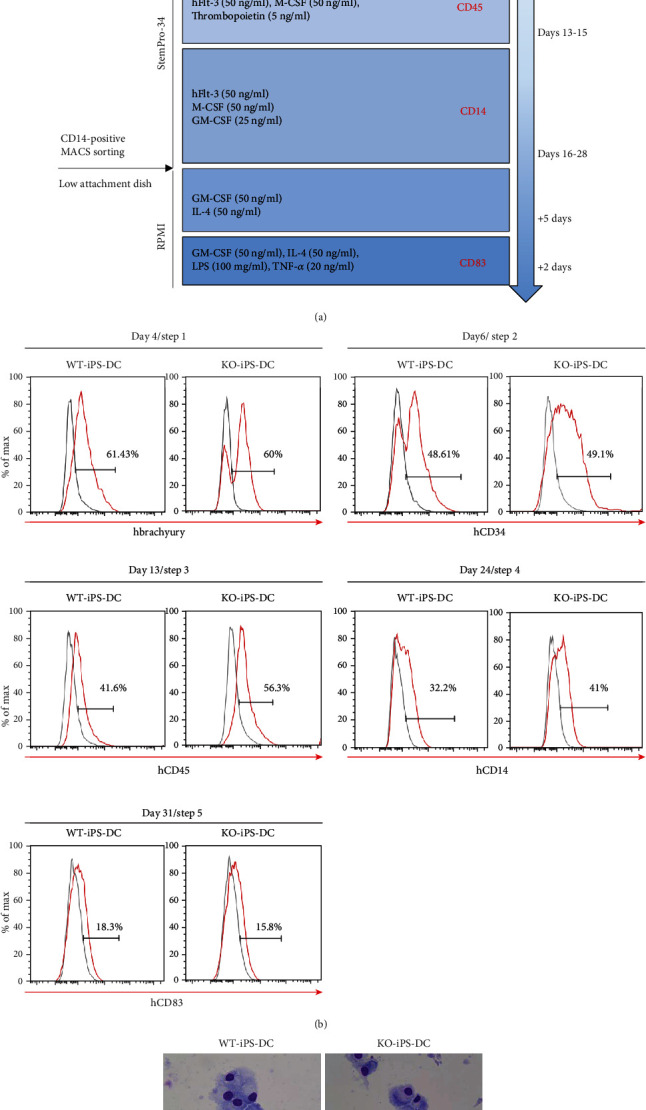
Differentiation of dendritic cells derived from wild-type and HLA DR knockout iPSCs. (a) Schematic representation of differentiation protocol for iPSCs into dendritic cells. (b) iPS cell-derived dendritic cells on day 4 in the 1^st^ step, day 6 in the 2^nd^ step, day 13 in the 3^rd^ step, day 24 in the 4^th^ step, and day 31 in the 5^th^ step were analyzed for the expression of brachyury, CD34, CD45, CD14, and CD83, respectively. (c) May-Grunwald-Giemsa staining of mature iPS induced DC on the glass slide is shown.

The differentiation of iPSCs to dendritic cells was divided into 5 steps (Figure 4(a)). Initially, we started to differentiate iPSCs into a broad mesodermal lineage and then more specifically into hematopoietic progenitors, monocyte-like cells, and finally matured dendritic cells. Based on this strategy, we initiated the differentiation of the mesodermal lineage by using BMP4, which has been reported to be an important cytokine for the initial stage of *in vitro* mesodermal commitment [17] of pluripotent stem cells [11, 19]. In the first step, we detected 60% of brachyury-positive cells (Figure 4(b)). Next, we treated VEGF, SCF, and bFGF to push cells towards a hemangioblast and mesodermal hematopoietic progenitor lineage and detected about 50% of the CD34+ hemangioblast-like population (Figure 4(b)). In step 3, we supplemented the cell culture media with hematopoietic cytokines on days 13~15 and obtained CD45-positive cells as 50% of the whole (Figure 4(b)). In step 4, the majority of cells were CD14+ monocyte-like cells. One month after differentiation from iPSCs (step 5), approximately 20% of cells were CD83+-matured dendritic cells (Figure 4(b)). These cells showed morphological resemblance to peripheral blood monocytes (Figure 4(c)). IFN-*γ* is secreted by innate immune cells soon after infection and stimulates DC, upregulating proinflammatory factors such as IL-12, IL-27, and TNF-*α* [20]. Interleukin 31 (IL-31) is a T cell-derived cytokine that signals via a heterodimeric receptor composed of an IL-31 receptor alpha (IL-31RA). IFN-*γ* is known as a stimulator for dendritic cells to increase the expression of IL-31RA and release proinflammatory mediators [21]. Therefore, we tested whether IFN-*γ* activated iPSC-derived DC to increase IL-12A, IL-27, and IL-31RA. As shown in Figure S8 A and B, iPSC-derived DC increased the expression of HLA DR, IL-12A, IL-27, and IL-31RA after treatment of interferon gamma.

Collectively, the differentiation efficiency of both wild-type and knockout iPSCs toward dendritic cells was very similar, and sufficient numbers of dendritic cells could be obtained from them.

### 3.5. Validation MLR Confirming the Absence of Immunogenicity of HLA DR Knockout iPSCs

**Figure 5:**
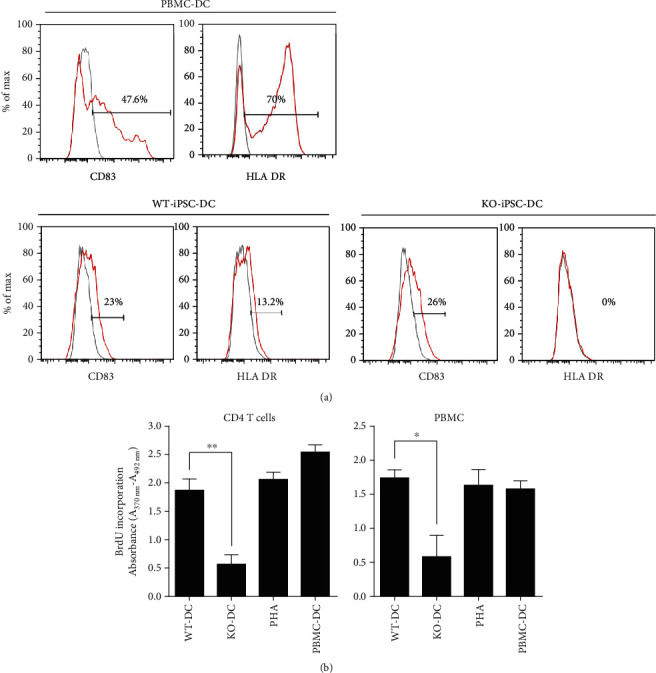
Validation of HLA DR knockout iPSC immunogenicity. CD83-positive matured DC from HLA DR knockout iPSCs does not express HLA DR, and there is no response to the CD4 T cells. (a) FACS analysis showed the expression of CD83 and HLA DR for wild-type iPSC-derived DC vs. HLA DR knockout iPSC-derived DC. (b) MLR assay revealed that wild-type iPSC-derived DC significantly induced allogeneic lymphocyte activation compared with HLA DR knockout iPSC-derived DC. (The asterisks indicate statistically significant changes: ^∗^*P* ≤ 0.5, ^∗∗^*P* ≤ 0.01, *N* = 3).

Next, we performed mixed lymphocyte reactions (MLR) to demonstrate the potential immune tolerance of HLA DR knockout iPSC-derived DCs. The CD83 level was not different between the wild-type and knockout DCs, while HLA DR was not detected only in the knockout cells (Figure 5(a)). We investigated whether wild-type iPSC-derived DC activated allogeneic naïve T cells while HLA DR knockout iPSC-derived DCs did not. Both DCs were cocultured with allogeneic naïve T cells. As shown in Figure 5(b), wild-type iPSC-derived DCs activated the proliferation of naïve T cells, but HLA DR knockout iPSC-derived DCs did not. As a control, we used PHA (phytohemagglutinin) and PBMC-derived DCs. They stimulated CD4 T cells and PBMC as much as wild-type iPSC-derived DCs (Figure 5(b)).

## 4. Discussion

Induced pluripotent stem cells (iPSCs), with their self-renewal and pluripotent characteristics, show promise in regenerative medicine, particularly for patients with severe degenerative diseases. A major merit of autologous iPSCs in clinical applications is the freedom from immune rejection. Although autologous transplantation is ideal from an immunological point of view, it is unlikely to be a standard therapy due to its high costs and long preparation time per patient. Another concern is the disease sensitivity of the donor cells from patients who have disease-specific genetic mutations [22]. Therefore, healthy donor-derived (allogeneic) cells may be better than patient-derived autologous cells.

One hurdle in the clinical application of allogeneic cells is the immune-mediated rejection of donor-derived cells by the recipients. Extensive effort has been devoted to the development of new strategies to induce immune tolerance of allogeneic transplants [23–25]. For successful allogeneic transplantation, donor-derived cells should not be recognized by host-derived T cells. In other words, the survival of an allograft bearing disparate human leukocyte antigens (HLAs) in an immunocompetent recipient depends on avoiding or overcoming an immune response to the infused cells. Therefore, the most effective approach to sustaining allograft survival is to eliminate mismatches in HLA between the donor and recipient.

HLA molecules are encoded by a large gene family and divided into classes I and II. Class I HLA (A, B, and C) presents intracellular peptides. For example, if the cell is infected by a virus, the HLA system brings fragments of the virus to the surface of the cell so that the cell can be destroyed by the immune system. These peptides are produced from digested proteins that are broken down in the proteasomes. Foreign antigens presented by MHC class I attract killer T cells (also called CD8-positive cells) that destroy cells. HLAs corresponding to MHC class II (DP, DM, DO, DQ, and DR) present extracellular antigens to T-lymphocytes. These particular antigens stimulate the multiplication of T-helper cells (also called CD4-positive T cells). They stimulate antibody-producing B cells to produce antibodies to that specific antigen.

Interestingly, many previous papers demonstrated that mismatches at the HLA DR (representative HLA class II) locus reveal the greatest impact on the development of an alloimmune response against transplanted organs such as the heart, kidney, and lung [2–7]. HLA DR are expressed constitutively in antigen-presenting cells, thymic epithelial cells, and endothelial cells. The HLA DR matching between recipients and a donor's microvascular endothelial cells may affect transplantation efficiency. For cell therapy, differentiated cells from iPSCs are transplanted into the lesion. Although these cells initially lack the expression of immune relevant molecules such as HLA DR, these cells express this molecule upon their administration *in vivo* [26]. The iPSC-derived cells operate within a microenvironment where these interact with stromal cells, growth factors, or extracellular matrix proteins and also face a variety of proinflammatory cytokines such as interferon *γ* and tumor necrosis factor alpha (IFN-*γ* and TNF-*α*). The microenvironment and its elements, together or independently, can modulate the expression of MHC II on these cells. It has been reported that IFN-*γ* induces HLA II expression in cardiac stem cells (CSC) [27], and IFN-*γ* plus TNF induce HLA class II in islet cells [28]. Therefore, HLA DR knockout iPSCs provide a novel cell source to overcome immune rejection in allogenic stem cell therapy.

The genome-editing technology has the power to not only correct genetic defects and fix diseases but also eliminate a specific gene locus, leading to the null function of the target gene. These effects are maintained permanently with a single treatment. Therefore, the gene editing method that can effectively target and specifically eliminate HLA DR will be a useful strategy in commercializing cell therapy. However, there are some disadvantages of genome-editing technology including TALEN and the CRISPR/Cas9 system. One of the common problems of these gene manipulation techniques is being off-target in that they miss their target in the genome [29]. Fortunately, it has been recently reported that the incidence of off-targets caused by these genome-editing techniques is very low in human stem cells compared with mouse embryonic stem cells [30, 31]. Therefore, the off-target issue can be controlled completely in the near future.

As proof of concept that transcription activator-like effector nucleases target HLA DR alleles for the complete and permanent attenuation of HLA DR expression, resulting in cells that can evade T cell recognition, we designed TALENs for gene editing in order to achieve selective elimination of the HLA DR expression. These completely disrupted the expression of HLA DR on not only fibroblasts but also HLA DR knockout iPSC-derived dendritic cells (Figures 2 and 5 and Supplementary Figure 4-6). Interestingly, the heterozygous clones of HLA DR knockout fibroblasts were activated by IFN-*γ*. They expressed HLA DR about 50% of wild type (Supplementary Figure 9). This suggested that haploid cells which contain the wild-type allele of HLA DR may be able to activate immune cells.

Dendritic cells (DCs) are specialized to regulate T cell response. They present antigenic peptides derived from the proteins in the context of major histocompatibility complex molecules to stimulate antigen-specific T cells. DCs have received an increasing amount of attention in cancer immunotherapy based on their abilities to modulate immunological responses; for example, DC-based cellular vaccination is being rapidly developed. Clinical trials of anticancer therapy with DC loaded with various cancer antigens have been conducted [32]. In most cases, the cell source of DC is monocytes from the patient by leukapheresis. However, the number of monocytes obtained from the peripheral blood is limited even with apheresis, and the potential of monocytes to differentiate into DC varies among the blood donors. The limitation of the cell source thus remains one of the major obstacles for the knockout iPSCs and developed the optimized differentiation protocol from iPSCs to DCs (Figure 4). iPSCs that can divide infinitely overcome limitations on the cell number, and DCs differentiated from HLA DR knockout iPSCs are useful in the field of cancer treatment using DCs.

The efficiency to generate iPSCs has been significantly improved by the use of small molecules, such as valproic acid, sodium butyrate, and vitamin C [10, 33, 34]. Since the p53 pathway has been known as a significant obstacle for reprogramming, the inhibition of the p53 pathways is a well-known strategy to increase the efficiency of iPSC generation [35, 36]. In this study, it was extremely difficult to generate iPSCs from HLA knockout fibroblasts. Thus, to improve the efficiency of reprogramming, we added small molecules and treated fibroblasts with 4 factors (Oct4, Sox2, Klf4, and c-Myc) after p53 siRNA transfection. However, we could not observe any single colony from HLA DR knockout HDF during 30 independent trials but once (Supplementary Table S1). For selecting HLA DR knockout cells, we performed negative sorting and single cell culture and generated iPSCs with HDFs passaged more than ten times. This implies that aged HDFs were used as a cell source for generating iPSCs and that the cells may have been senescent and with low proliferation rates. This may be the reason why HLA DR knockout iPSC generation was challenging.

**Figure 6:**
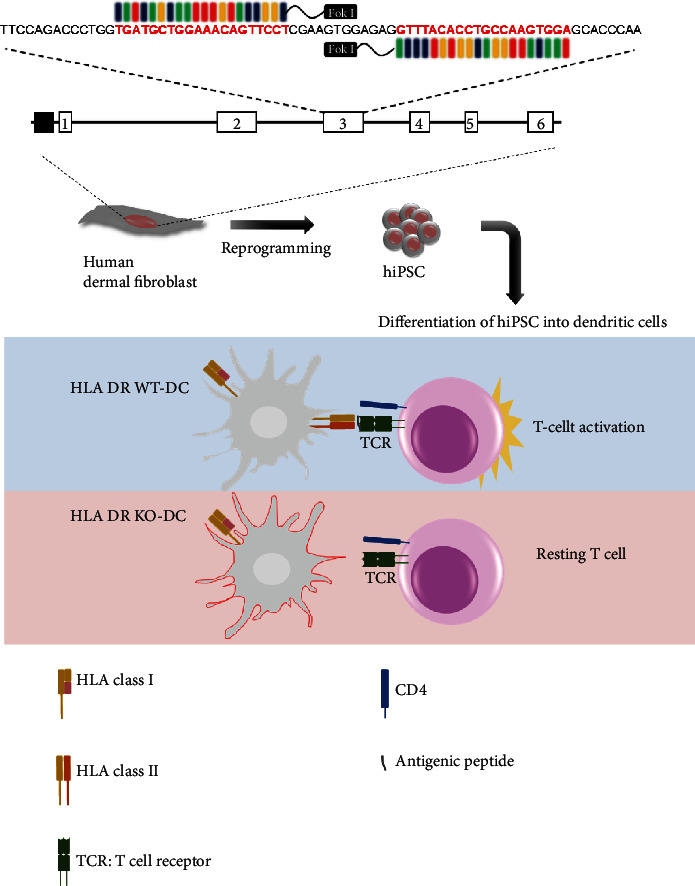
Establishment of universally compatible immune nonresponsive human iPSCs by genome editing. Mismatches at the HLA DR (representative HLA class II) locus reveal the greatest impact on the development of an alloimmune response against transplanted organs such as the heart, kidney, and lung. Transcription activator-like effector nucleases (TALENs) were designed for selective removal of HLA DR expression. The TALENSs completely disrupted the expression of HLA DR on human dermal fibroblast cells (HDFs). Dendritic cells derived (differentiated) from HLA DR knockout iPSCs did not express HLA DR and reduced CD4^+^ T cell activation. These engineered iPSCs may have resolved the problem of immune rejection and provided a novel clinical application of derivatives.

In summary, we demonstrated that human dermal fibroblasts could be permanently modified by TALEN to eliminate HLA DR expression. This elimination was maintained after iPSCs were generated, and also, the HLA DR expression was not detected in iPSC-derived dendritic cells. Since the HLA DR knockout iPSC-derived dendritic cells did not elicit a response from CD4-positive T cells or peripheral blood, these cells obtained immune tolerance and may be a very useful cell source for immune cell therapy. In Figure 6, we summarized the process that establishes universally compatible immune nonresponsive human iPSCs by genome editing. HLA DR knockout iPSCs did not show any difference from wild-type iPSCs in terms of self-renewal and differentiation potential, indicating that HLA DR knockout iPSCs can be expanded infinitely and applied to various types of diseases after differentiating into the target cells whenever necessary.

In this study, we demonstrated that TALEN-mediated genome editing successfully generated induced pluripotent cells without HLA DR expression and that dendritic cells differentiated from these iPSCs displayed immune tolerance. Therefore, clinical grade iPSCs from a healthy donor after a single allogeneic mutation could be applied into multiple recipients, which would be a significant stride towards off-the-shelf stem cell therapy using universal cells that can be predeployed at multiple sites and infused to multiple recipients when needed.

## 5. Conclusion

Here, we successfully generated induced pluripotent cells without HLA DR expression using the TALEN system. In addition, dendritic cells differentiated from these iPSCs displayed T cell immune response. We hope to help minimize the risk of rejection this proof-of-concept study into a possible future therapy.

## Data Availability

All data used to support the findings of this study are included within the article. And those are available from the corresponding author upon request.

## Supplementary Materials

Supplementary Materials Supplementary Figure S1: T7E1 assay showed TALEN-induced mutation in fibroblasts. Supplementary Figure S2: the introduction of the reporter system. It consists of the mRFP gene, the programmable nuclease's target sequence (left and right half-sites), and the eGFP gene. Supplementary Figure S3: fluorescent microscopy of HEK293T cells cotransfected with reporter plasmid and TALENs. Supplementary Figure S4–6: loss of HLA DR expression on 4 different clones of HLA DR knockout fibroblasts after genome editing with TALENs. Supplementary Figure S7: expression of HLA class I and HLA DR in WT-iPS and HLA DR KO-iPS cells treated with various IFN-*γ* concentrations. Supplementary Figure S8: expression of HLA class I and HLA DR in WT-iPS and HLA DR KO-iPS cells treated with various IFN-*γ* concentrations. Both cells expressed HLA class I but did not express HLA DR after treatment of IFN-*γ*. Supplementary Figure S9: HLA DR expression on clones of HLA DR hetero-knockout fibroblasts after genome editing with TALENs. (A) Levels of HLA DR genetic disruption determined by the T7E1 assay and alignment of the genomic sequences of both alleles at the TALEN recognition site. Allele #1 shows the 2 bp deletion, and protein sequences are completely different with Allele #2. (B) HLA DR protein expressions in wild-type and HLA DR hetero-knockout fibroblasts were compared by FACS analysis. Supplementary Table S1: list of protocols used to generate human iPSCs from HLA DR KO fibroblasts.
