# Supplementary material for: HLA DR Genome Editing with TALENs in Human iPSCs Produced Immune-Tolerant Dendritic Cells
Source: Stem Cells Int. 2021 May 20;2021:8873383. doi: 10.1155/2021/8873383 (PMC8163544; doi:10.1155/2021/8873383)
Supplement: Supplementary Materials — Supplementary Figure S1: T7E1 assay showed TALEN-induced mutation in fibroblasts. Supplementary Figure S2: the introduction of the reporter system. It consists of the mRFP gene, the programmable nuclease's target sequence (left and right half-sites), and the eGFP gene. Supplementary Figure S3: fluorescent microscopy of HEK293T cells cotransfected with reporter plasmid and TALENs. Supplementary Figure S4–6: loss of HLA DR expression on 4 different clones of HLA DR knockout fibroblasts after genome editing with TALENs. Supplementary Figure S7: expression of HLA class I and HLA DR in WT-iPS and HLA DR KO-iPS cells treated with various IFN-γ concentrations. Supplementary Figure S8: expression of HLA class I and HLA DR in WT-iPS and HLA DR KO-iPS cells treated with various IFN-γ concentrations. Both cells expressed HLA class I but did not express HLA DR after treatment of IFN-γ. Supplementary Figure S9: HLA DR expression on clones of HLA DR hetero-knockout fibroblasts after genome editing with TALENs. (A) Levels of HLA DR genetic disruption determined by the T7E1 assay and alignment of the genomic sequences of both alleles at the TALEN recognition site. Allele #1 shows the 2 bp deletion, and protein sequences are completely different with Allele #2. (B) HLA DR protein expressions in wild-type and HLA DR hetero-knockout fibroblasts were compared by FACS analysis. Supplementary Table S1: list of protocols used to generate human iPSCs from HLA DR KO fibroblasts. [file 8873383.f1.pdf]

Supplementary Information

Supplement Figure 1

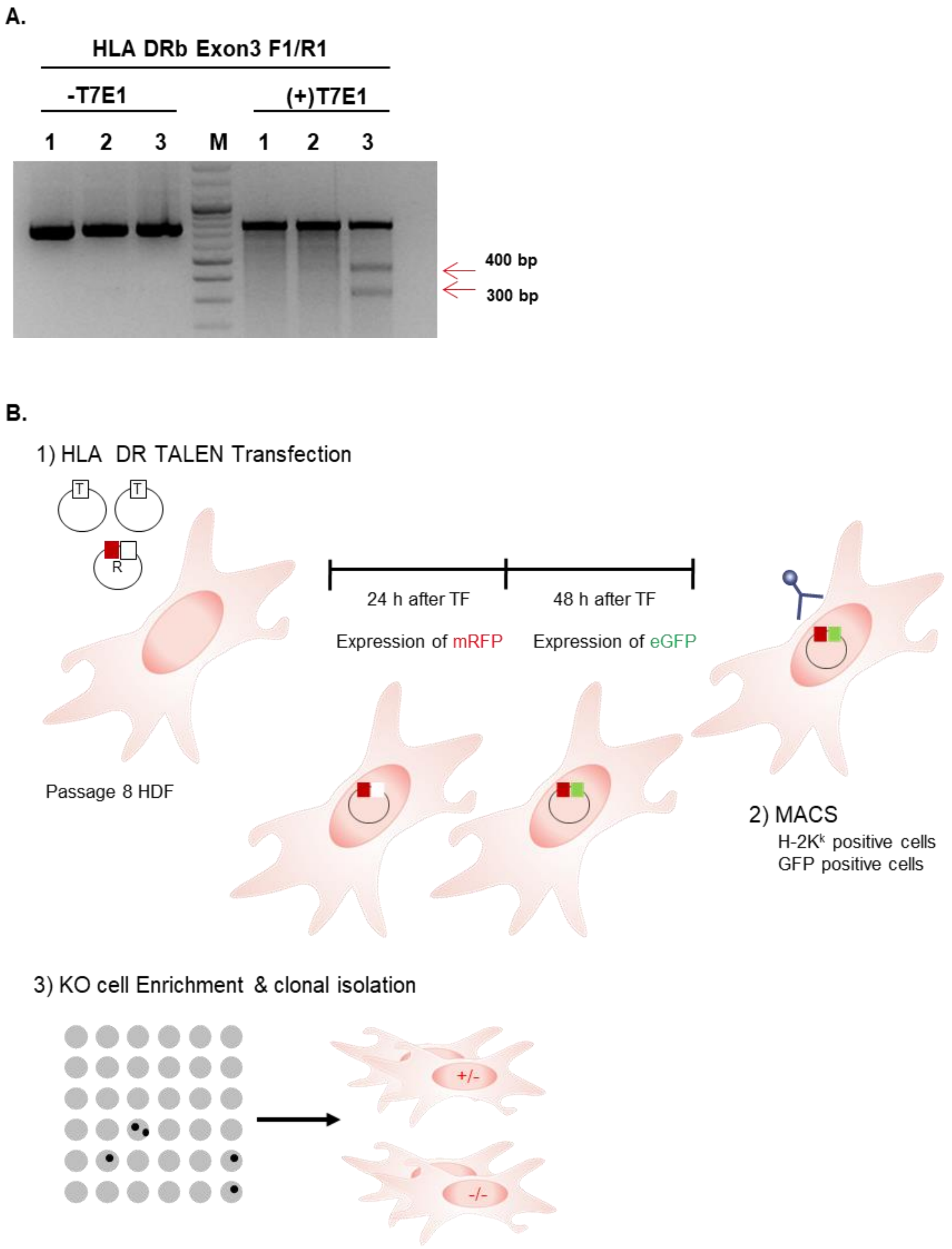

Supplementary Figure S1. (A) One of three TALEN pairs could effectively target HLA DR, confirmed by T7E1 assay. T7E1 assay showed TALEN induced mutation in fibroblast. Arrow

indicates the expected positions of cleaved DNA bands. (B) The working mechanism of the H-2K^k magnetic reporter. A schematic illustrating the enrichment of mutant cells.

1) mRFP is constitutively expressed, but eGFP and H-2K^k are expressed by the activity of engineered nucleases. 2) H-2Kk-expressing cells can be magnetically separated using anti-H-2kk antibody conjugated to magnetic beads. Mutant cells were enriched in this population of H-2kk-expressing cells. 3) To obtain single-cell clones from the TALEN-modified HDF pool, we performed single cell culture after sorting with magnetic separation.

Supplement Figure 2

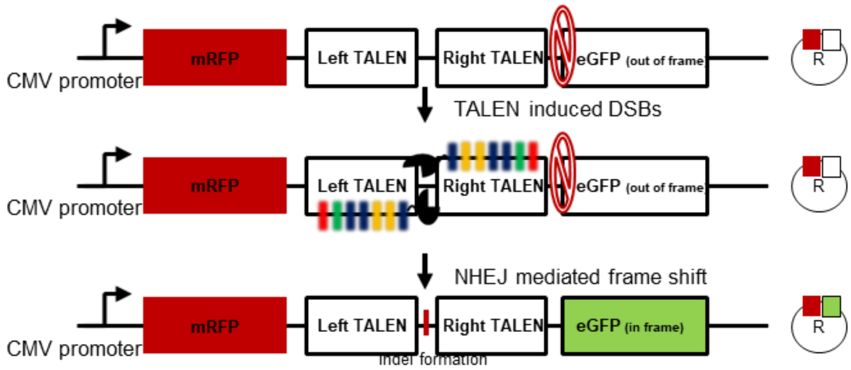

Supplementary Figure S2. The reporter consists of the mRFP gene, the programmable nuclease's target sequence (left and right half-sites) and the eGFP gene. mRFP is constitutively expressed from the CMV promoter, whereas functional eGFP is not expressed because its sequence is out of frame in the absence of programmable nuclease activity. When a double-strand break is introduced into the target sequence by programmable nucleases, the break is repaired by nonhomologous end-joining (NHEJ), which often causes frameshift mutations. Such mutations can render eGFP in frame with mRFP, inducing the expression of the mRFP-eGFP fusion protein.

Supplementary Figure 3

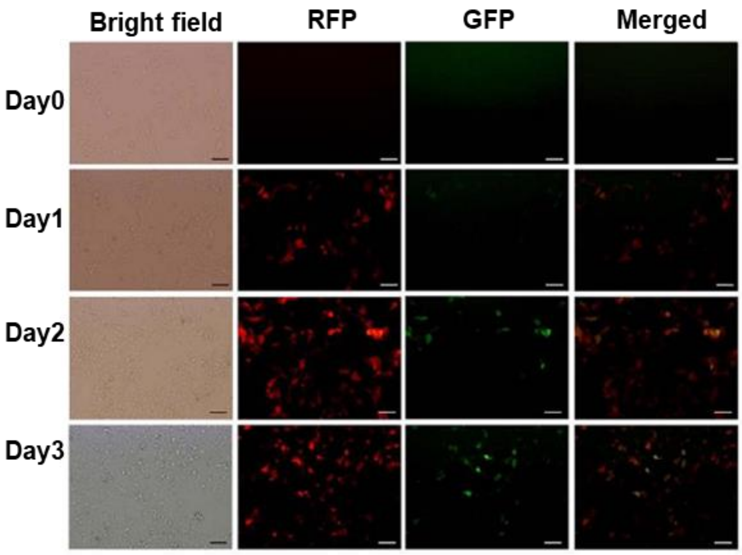

Supplementary Figure S3. Fluorescent microscopy of HEK293T cells co-transfected with reporter plasmid and TALENs

Supplement Figure 4

A.

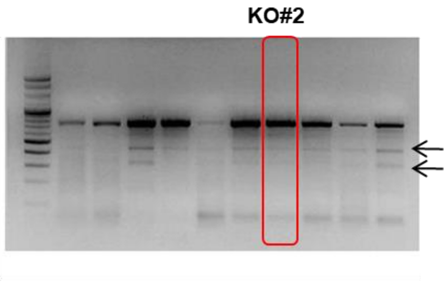

B.

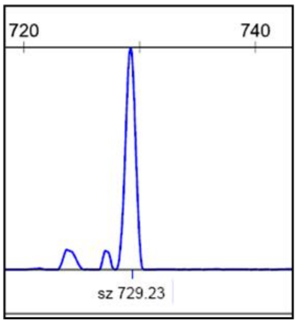

C.

		Protein amino acid
WT	TGATGCTGGAAACAGTTCCT CGGAGTGGAGAG GTTTACACCTGCCAAGTGGA	TVPRSGEVYTCQVEHPSVT
KO#2	TGATGCTGGAAACAGTTCCT CGAAGT-GAGAG GTTTACACCTGCCAAGTGGA	TVPRSERFTPAKWSTQA
	TGATGCTGGAAACAGTTCCT CGA--- GGAGAG GTTTACACCTGCCAAGTGGA	TVPR- GEVYTC -stop-

D.

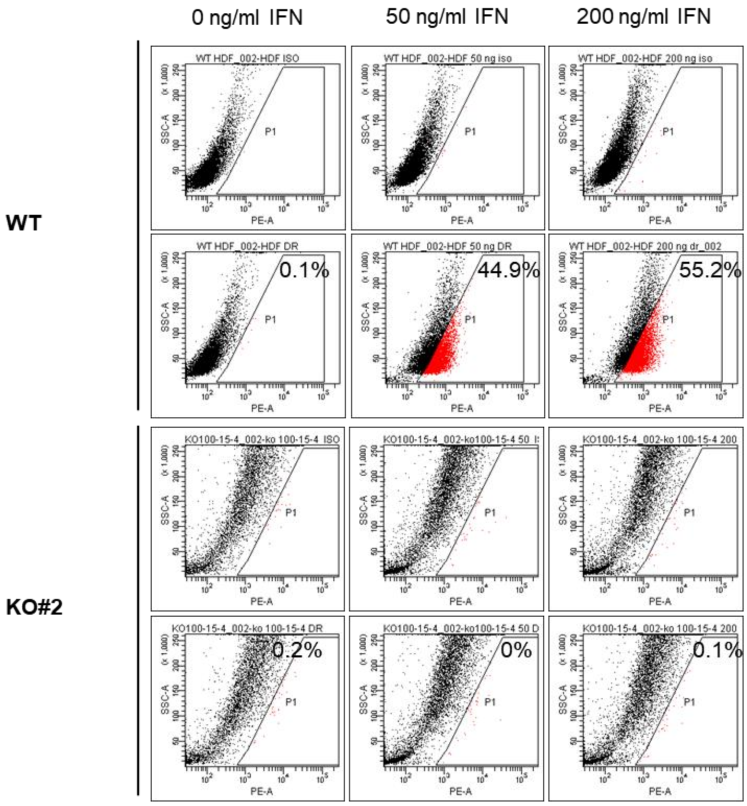

Supplement Figure 5

A.

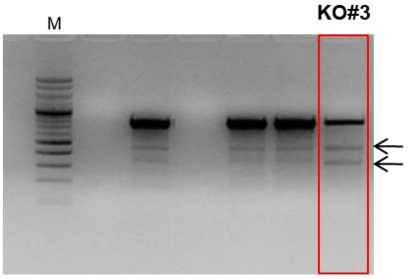

B.

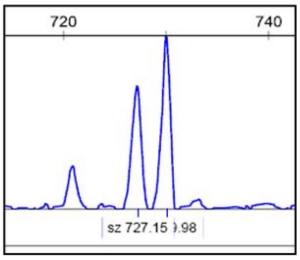

C.

Protein amino acid

WT TGATGCTGGAAACAGTTCCT CGGAGTGGAGAG GTTTACACCTGCCAAGTGGA TVPRSGEVYTCQVEHPSVT

KO#3 TGATGCTGGAAACAGTTCCT CGAAGTG-AGAG GTTTACACCTGCCAAGTGGA TVPRR -EVYTC
TGATGCTGGAAACAGTTCCT CGAAG---AGAG GTTTACACCTGCCAAGTGGA TVPRSERFTPAKWSTQA
-stop-

D.

0 ng/ml IFN | 50 ng/ml IFN | 200 ng/ml IFN

WT

KO#3

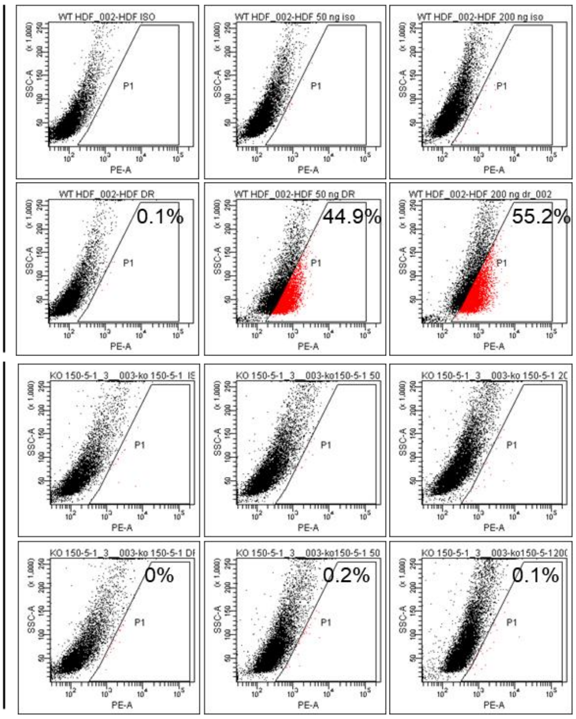

Supplement Figure 6

A.

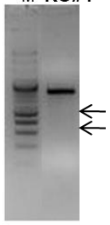

B.

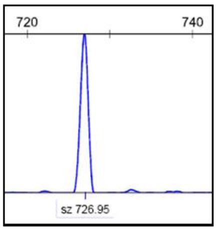

C.

		Protein amino acid
WT	TGATGCTGGAAACAGTTCCT CGGAGTGGAGAG GTTTACACCTGCCAAGTGGA	SGEVYTCQVEHP
KO#4	TGATGCTGGAAACAGTTCCT CGA----GAGAG GTTTACACCTGCCAAGTGGA	

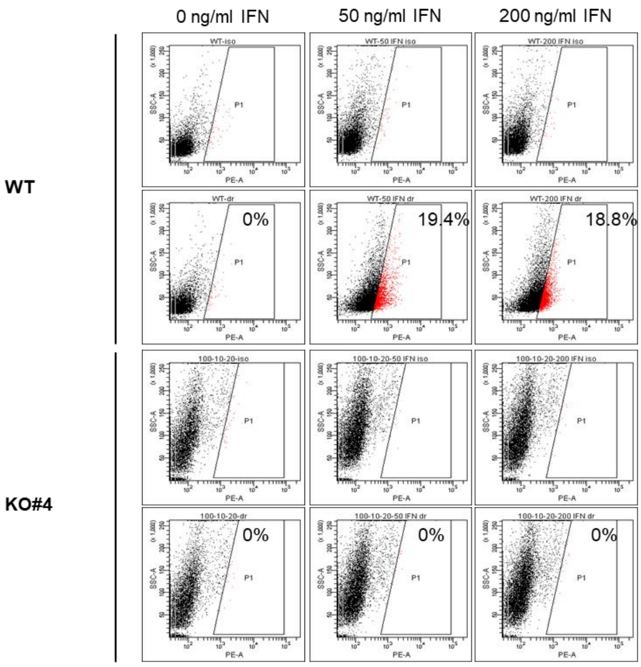

Supplementary Figure S4-6. Loss of HLA DR expression on 4 different clones of HLA DR knockout fibroblasts after genome editing with TALENs. Clone name of KO#2; 150-5-1, Clone name of KO#3; 100-15-4 and Clone name of KO#4; 100-10-2. (A) Levels of HLA DR genetic disruption determined by the T7E1 assay. (B) Different fluorescent intensity of wild type and mutant fibroblasts were shown after transfection of the TALEN encoding plasmids. (C) Alignment of the genomic sequences of wild type and mutant clone at the TALEN recognition site. (D) HLA DR protein expression in wild type and HLA DR mutant fibroblasts were compared by FACS analysis

Supplement Figure 7

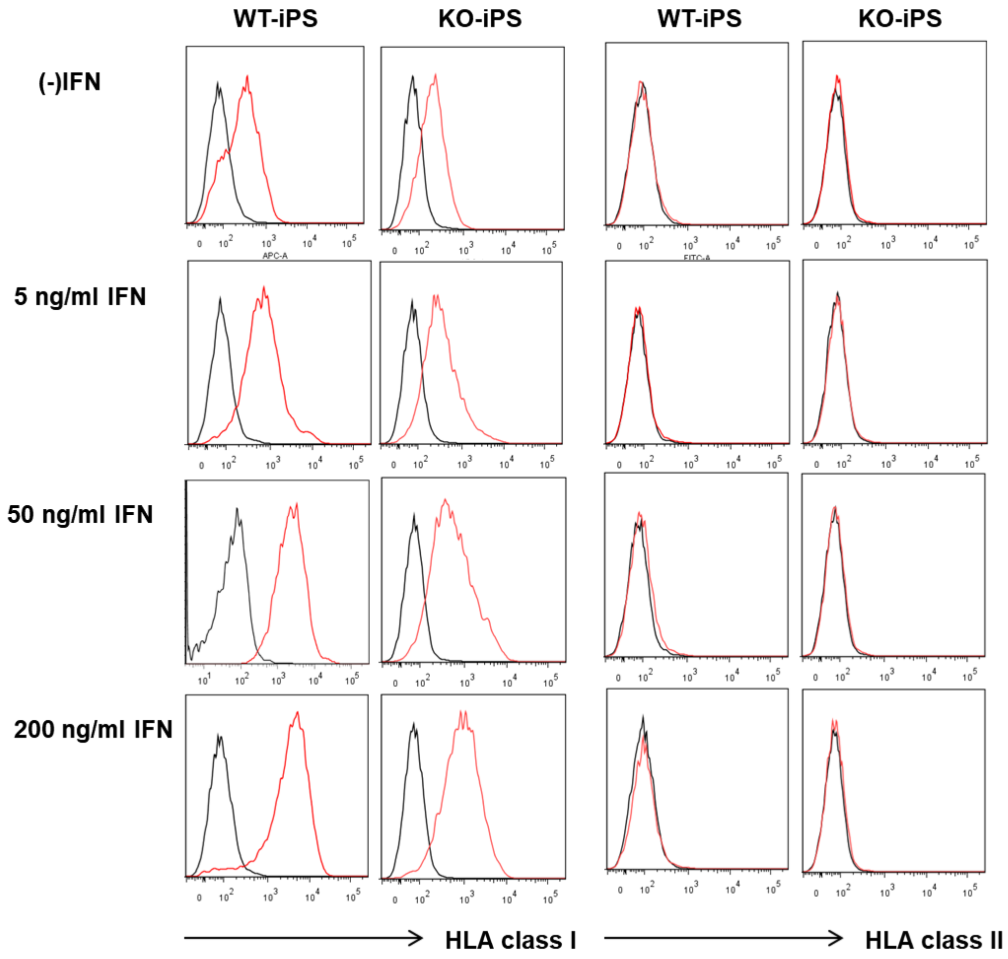

Supplementary Figure S8. Expression of HLA Class Ⅰ and HLA DR in WT-iPS and HLA DR KO-iPS cells treated with various IFNγ concentration. Both cells expressed HLA class I but did not express HLA DR after treatment of IFNγ.

Supplement Figure 8

A.

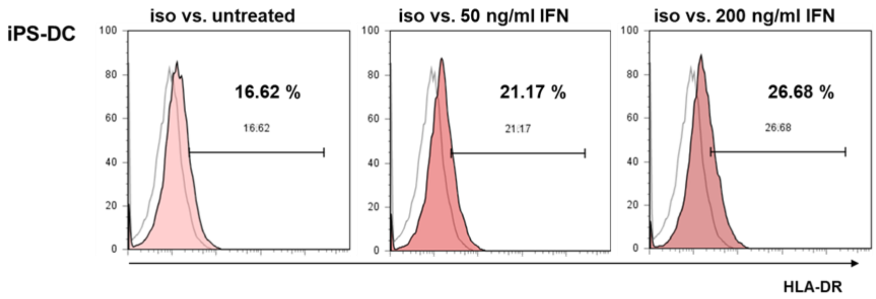

B.

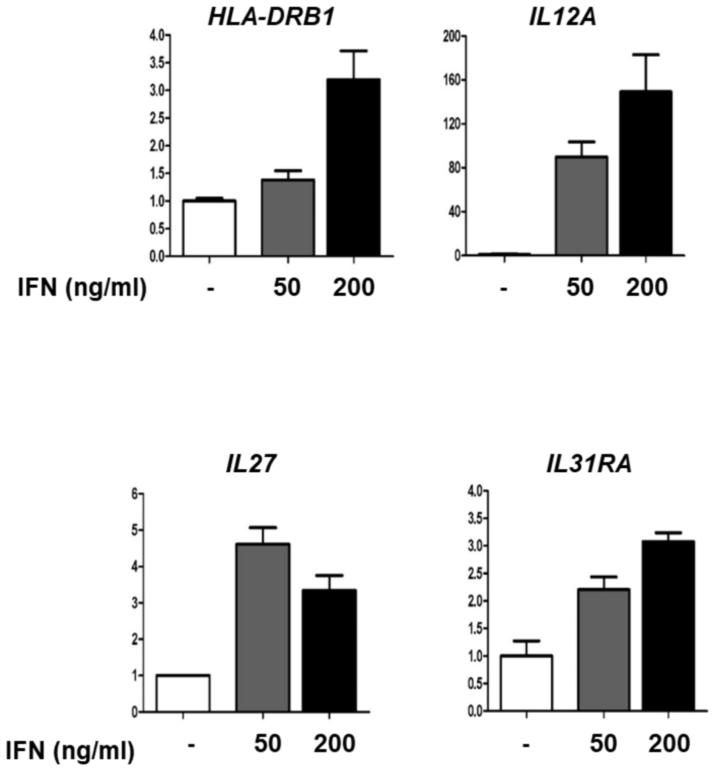

Supplement Figure 9

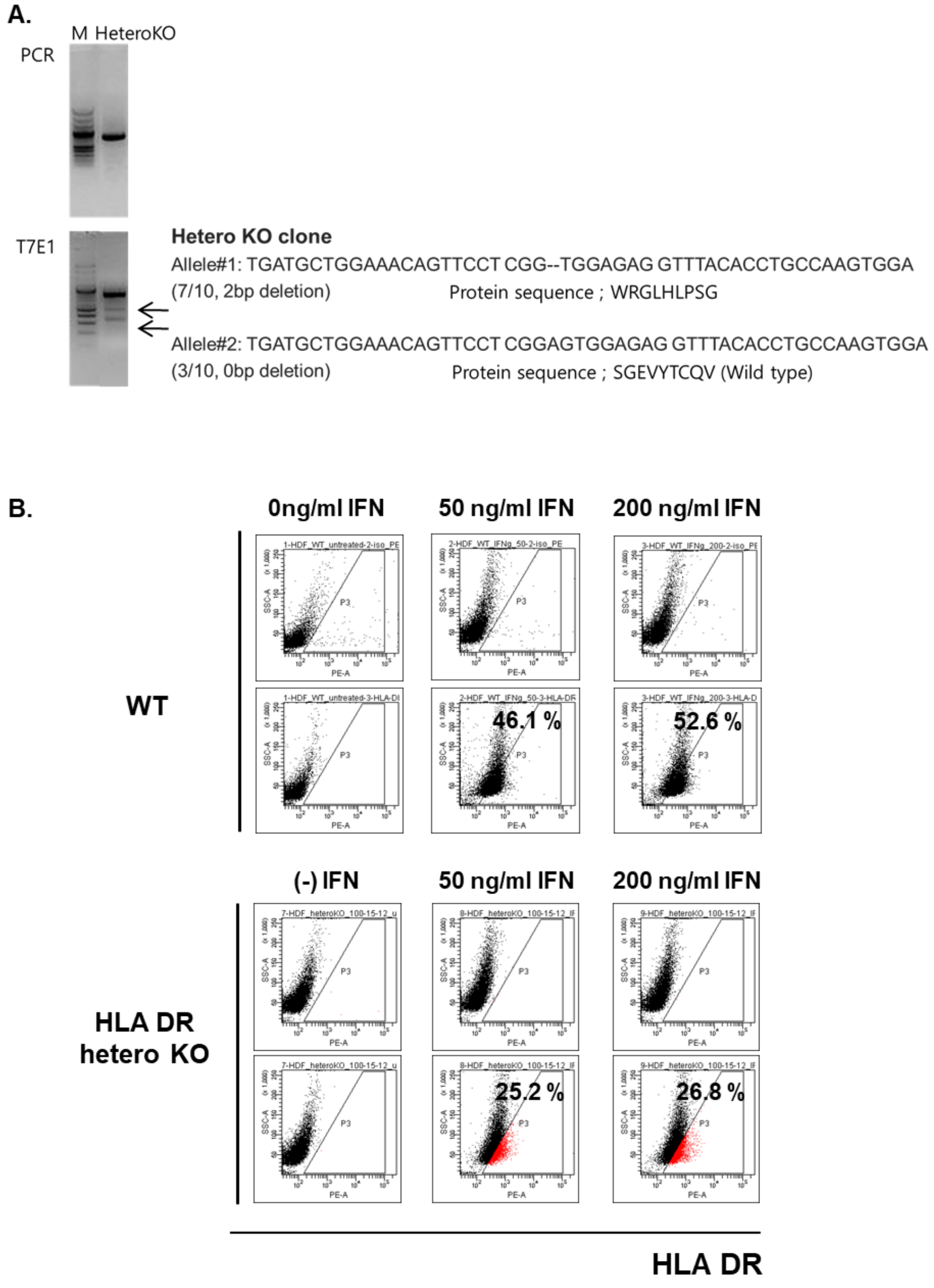

Supplementary Figure S8. HLA DR expression on clones of HLA DR hetero-knockout fibroblasts after genome editing with TALENs. (A) Levels of HLA DR genetic disruption determined by the T7E1 assay and alignment of the genomic sequences of both alleles at the

TALEN recognition site. Allele #1 shows the 2bp deletion and protein sequences are completely different with Allele #2. (B) HLA DR protein expression in wild type and HLA DR hetero-knockout fibroblasts were compared by FACS analysis

Supplementary Table S1. List of protocols used to generate human iPSCs from HLA DR KO fibroblasts.

Cell type	Genome engineering		Factor	iPSC generation		
	Gene_tool	Clone		Success	Method	Trial
HDF	Normal		OSKM#	o	Yamanaka protocol	10
HDF	HLA DR_TALEN	KO#1	OSKM	X	Yamanaka protocol	1
HDF	HLA DR_TALEN	KO#1	OSKM	X	Increase viral plasmid conc.	1
HDF	HLA DR_TALEN	KO#1	OSKM	X	Seeding cell number	1
HDF	HLA DR_TALEN	KO#1	OSKM	X	2nd virus infection	1
HDF	HLA DR_TALEN	KO#1	OSKM	X	3rd virus infection	1
HDF	Normal		OSKM	o	Vc+VPA*	3
HDF	HLA DR_TALEN	KO#1	OSKM	o	Vc+VPA	2
HDF	HLA DR_TALEN	KO#1	OSKM	X	Vc+VPA	3
HDF	HLA DR_TALEN	KO#1	OSKM	X	P53 knock down	1
HDF	HLA DR_TALEN	KO#1	OSKM	X	P53 knock down +VPA	2
HDF	HLA DR_TALEN	KO#2	OSKM	X	MET enhancing reagent	1
HDF	HLA DR_TALEN	KO#2	OSKM	X	Vc+VPA	2
HDF	HLA DR_TALEN	KO#2	OSKM	X	Vc+VPA	1
HDF	HLA DR_TALEN	KO#3	OSKM	X	Vc+VPA	1
HDF	HLA DR_TALEN	KO#3	OSKM	X	Matrigel + Vc+VPA+A89-01	2
HDF	HLA DR_TALEN	KO#3	OSKM	X	Matrigel + Vc+VPA+mTeSR media	1
HDF	HLA DR_TALEN	KO#3	OSKM	X	Matrigel + Vc+VPA+E7 media	3
HDF	HLA DR_TALEN	KO#2	OSKM	X	Matrigel + Vc+VPA+E7 media	1

* : VC :Vitamin C, VPA : Valproic acid

# : O :OCT4, S:SOX2, K:KLF4, M:c-MYC
